# Seroprevalence of HIV among pregnant women in Ethiopia: a systematic review and meta-analysis

**DOI:** 10.1186/s13104-018-4022-1

**Published:** 2018-12-19

**Authors:** Demeke Geremew, Fitsumbrhan Tajebe, Sintayehu Ambachew, Aklilu Endalamaw, Setegn Eshetie

**Affiliations:** 1 Department of Immunology and Molecular Biology, School of Biomedical and Laboratory Sciences, University of Gondar, P.o.Box: 196, Gondar, Ethiopia; 2 Department of Clinical Chemistry, School of Biomedical and Laboratory Sciences, University of Gondar, Gondar, Ethiopia; 3 Department of Pediatrics and Child Health Nursing, School of Nursing, College of Medicine and Health Sciences, University of Gondar, Gondar, Ethiopia; 4 Department of Medical Microbiology, School of Biomedical and Laboratory Sciences, University of Gondar, Gondar, Ethiopia

**Keywords:** HIV, Pregnant women, Systematic review, Meta-analysis, Ethiopia

## Abstract

**Objective:**

This systematic review and meta-analysis aimed to determine the pooled prevalence of HIV among pregnant women in Ethiopia.

**Result:**

PubMed, EMBASE, Science Direct and Google scholar databases were searched to retrieve 15 relevant articles based on the inclusion criteria. A total of 13,746 participants were included in the original studies and considered in this analysis. Among subjects, 717 were infected with HIV only, and 12 were HIV-HBV co-infected pregnant women. In this meta-analysis, the pooled prevalence of HIV among pregnant women in Ethiopia was 5.74% (95% CI 3.96–7.53%). Regional analysis showed that 9.50% (95% CI 7.76–11.23%) in Amhara, 4.80% (95% CI 3.12–6.49%) in Addis Ababa, 2.14% (95% CI − 0.54 to 4.82%) in SNNP and 4.48% (95% CI 2.56–6.41%) in Oromia region. Besides, six studies reported HIV-HBV co-infection and the pooled prevalence was 0.68% (95% CI 0.27–1.08%) among pregnant women in Ethiopia.

**Electronic supplementary material:**

The online version of this article (10.1186/s13104-018-4022-1) contains supplementary material, which is available to authorized users.

## Introduction

Globally, about 17.8 million people living with HIV were women’s above 15 years old [1]. According to UNAIDS 2017 country fact sheet, Ethiopia has 710,000 people living with HIV. Out of whom 400,000 were women above 15 years old [2]. Young women from 15 to 24 years age group have eight times higher chance of getting HIV infection related to their corresponding male counterparts [3].

Screening pregnant women for HIV is essential to initiate early antiretroviral therapy (ART) that improves maternal health and decrease the risk of HIV mother to child transmission (MTCT). As a result, women who are HIV negative during antenatal screening are recommended for re-testing in the third trimester, or during labor or shortly after delivery [4]. MTCT of HIV is a significant contributor to the HIV pandemic, accounting for 9% of new infections globally. Effective prevention of MTCT can reduce the risk of vertical transmission from 15 to 45% to below 5% during breastfeeding. However, only 69% of HIV seropositive pregnant women received ART in Ethiopia [5, 6] suggesting the existence of unaddressed issue in the country. Therefore, the emergence of HIV free generation is in question if we are unable to preclude HIV infection in pregnant women [3].

The magnitude of HIV among pregnant women attending antenatal clinic (ANC) in Ethiopia varies from 0.2% [7] to 12.1% [8]. Moreover, nationwide HIV sentinel survey reported 3.0% in 2009 [9] and 2.2% in 2014 [10]. Nevertheless, through robust screening and use of sensitive and specific laboratory tests, the burden could be much higher than the stated one. Hence, evidence regarding the exact magnitude of most prevailing infections like HIV is required to guide public health intervention and controls. Therefore, the purpose of this systematic review and meta-analysis was to assess the pooled prevalence of HIV among pregnant women in Ethiopia.

## Main text

### Methods

#### Study protocol registration

This study was registered in PROSPERO database with protocol number, CRD42018088593.

#### Search strategy

We made an inclusive literature search from PubMed, EMBASE, Science Direct and Google scholar databases based on Preferred Reporting Items for Systematic Reviews and Meta-Analyses (PRISMA) statement [11]. The following key words was used for PubMed database searching; [“HIV” OR “human immunodeficiency virus” AND (seroprevalence OR prevalence) AND “pregnant women” OR “pregnant” AND “Ethiopia”]. Besides, grey literatures and reference lists of relevant articles were also retrieved to find additional studies.

#### Eligibility criteria

All articles fulfilling the following conditions were screened and subsequently assessed for eligibility. Studies with cross sectional and prospective cohort study design, conducted only in Ethiopia and reporting seroprevalence of HIV among pregnant women, published in English language up to December 31, 2017. Studies with a clear description of participants involved and number of participants tested for HIV, number and/or prevalence of HIV cases, and state the region of Ethiopia at which the research was conducted were also considered. Nevertheless, review articles, conference abstracts, sentinel and case reports were excluded.

#### Study selection, quality assessment and data extraction

The full texts of articles which are relevant by title and abstract were thoroughly reviewed for eligibility.

The quality of included studies was evaluated by using Joanna Brigg’s Institute (JBI) quality assessment checklist for prevalence studies [12]. Based on the JBI checklist, studies with a quality score of 50% and above were considered as high quality and involved in the analysis (Additional file 1).

Extracted data includes the following descriptive information: Author and year of publication, study area/region, study design, sample size, mean age of participants, screening method used, number of HIV cases and prevalence rate. Two independent reviewers (DG and SE) were involved in study selection, quality assessment and data extraction. Disagreement between the reviewers was solved by discussion.

#### Data analysis

Extracted data were entered into a table using Microsoft Excel and exported into Stata version 11.0 (StataCorp, College Station, TX, USA) for analysis. The I^2^ statistics was used to assess heterogeneity between studies. I^2^ ≥ 50% was considered as statistically significant [13]. Possible sources of variation were explored using subgroup analysis. In pooled prevalence analysis and 95% confidence intervals (95% CIs), the random effects model (DerSimonian-Laird method) [14] was used. The overall and subgroup analysis of random effects model with 95% CIs were calculated and demonstrated using a forest plot.

The existence of publication bias was determined using Egger’s test (a statistical analogue for funnel plot). Egger’s test, p < 0.05 was considered as statistically significant [15]. The effect of each study on the overall pooled prevalence was determined by using sensitivity analysis (Additional file 2). Sensitivity test eliminates each study step by step in the analysis to indicate the pooled effect sizes and related heterogeneity attributed by each individual study.

### Results

#### Study documentation and retrieval

**Fig. 1:**
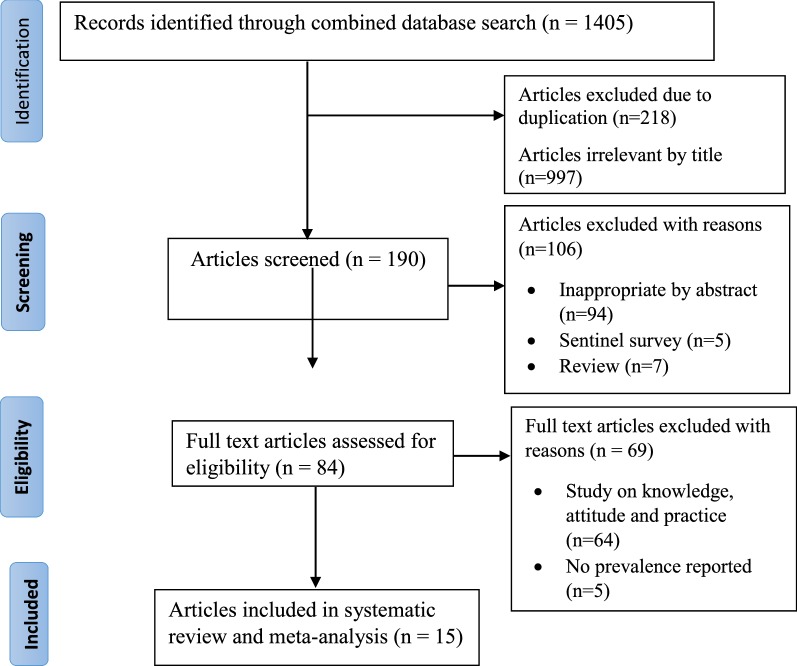
PRISMA flow chart for the studies screened, reviewed and included

After a combined literature search, 15 studies were eligible for inclusion in meta-analysis (Fig. 1).

Thirteen studies used rapid HIV-1/HIV-2 test kits for HIV screening as per the national algorithm for HIV testing and two studies employed ELISA. Among the 15 studies [7, 8, 16–28] included, a total of 13,746 pregnant women were screened for HIV in four different regions of Ethiopia. Regional distribution of studies revealed that five [17, 21, 23, 25, 28] from Amhara, two [20, 22] from Addis Ababa, three [7, 24, 26] from southern nations nationalities and peoples (SNNP) of Ethiopia and five [8, 16, 18, 19, 27] from Oromia region. Nevertheless, there was no any study from other regions of Ethiopia fulfilling the inclusion criteria. Except one prospective cohort study, all studies were cross sectional with study participants ranging from 165 in SNNP [26] to 7817 in Oromia [18] and conducted from 2002 to 2017. Only about six studies [19, 20, 22, 24, 26, 28] reported HIV-HBV co-infection. The rate of MTCT of HIV was determined in none of the involved studies.

The mean age of the participants were ranging from 24 to 26.1 years old. Out of 13, 746 participants screened for HIV, 717 were confirmed to be HIV seropositive. Out of 717 HIV seropositive pregnant women, 164 were from Amhara, 30 were from Addis Ababa, 18 were from SNNP, and 505 were from Oromia region. Of 717 HIV seropositive pregnant women, 12 of them were co-infected with hepatitis B virus (HBV) (Additional file 1).

#### Pooled HIV prevalence among pregnant women

**Fig. 2:**
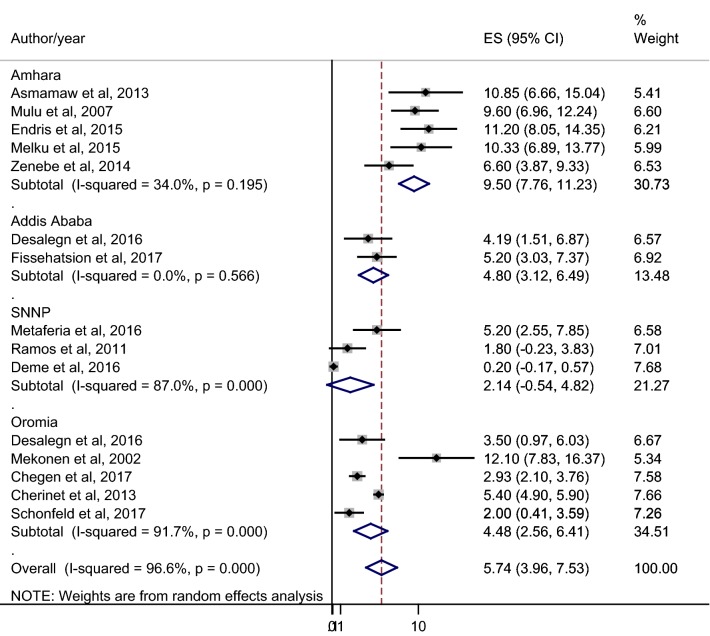
Pooled prevalence estimate (ES) of HIV among pregnant women in Ethiopia. The midpoint and the length of each segment indicated prevalence and a 95% CIs respectively whereas the diamond shape showed the combined prevalence

The overall pooled prevalence of HIV among pregnant women in Ethiopia from the random effects model was 5.74% (95% CI 3.96–7.53: I^2^ = 96.6%: Egger’s test, p = 0.06). Specifically, subgroup analysis based on different regions of Ethiopia revealed that 9.50% (95% CI 7.76–11.23) in Amhara, 4.80% (95% CI 3.12–6.49) in Addis Ababa, 2.14% (95% CI − 0.54 to 4.82) in SNNP and 4.48% (95% CI 2.56–6.41) in Oromia region (Fig. 2).

#### Pooled prevalence of HIV-HBV co-infection among pregnant women

**Fig. 3:**
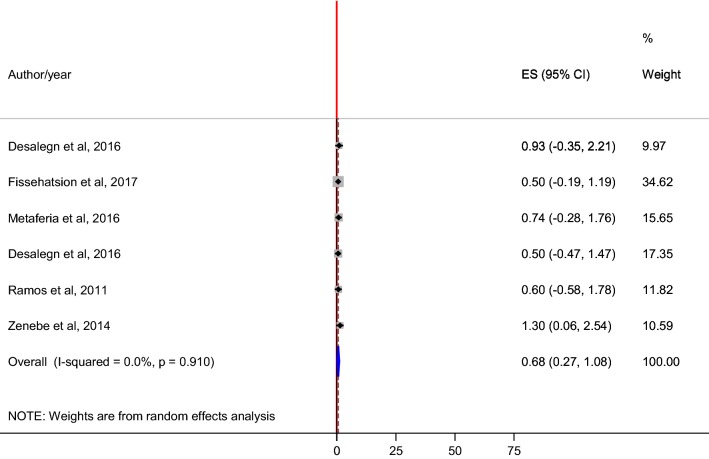
Pooled prevalence estimate (ES) of HIV-HBV co-infection among pregnant women in Ethiopia. The midpoint and the length of each segment indicated prevalence and a 95% CIs respectively whereas the diamond shape showed the combined prevalence

The overall pooled prevalence of HIV-HBV co-infection among pregnant women in this meta-analysis was 0.68% (95% CI 0.27–1.08) (Fig. 3).

### Discussion

HIV infection is the significant cause of morbidity and mortality particularly in resource limited countries including Ethiopia. Other sexually transmitted infections (STIs) such as HBV infection is also commonly encountered in these groups of patients, because of shared transmission ways and geographical incidence [29]. Therefore, to reduce the rapidly growing burden of HIV and HIV-HBV co-infections in Ethiopia, it is very important to protect young women and decrease the rate of vertical transmission and protect future generations [30]. As yet, there is no evidence of pooled HIV and HIV-HBV co-infection estimates among pregnant women in Ethiopia. Therefore, this meta-analysis was the first of its kind to determine the pooled estimates of the diseases burden among pregnant women.

Based on this meta-analysis, the overall pooled prevalence of HIV in pregnant women in Ethiopia was 5.74% (95% CI 3.96–7.53%). This pooled estimate is five times higher than the national HIV prevalence among the general population of Ethiopia, 1.14% [31]. An increased burden of HIV in ANC in this meta-analysis might be more likely multi-factorial. First, the mean age of the participants involved in this meta-analysis was 24 to 26 years, indicating all women in ANC were sexually active age groups [32]. Second, general population includes men who have lower HIV prevalence compared to pregnant women [32, 33]. Finally, the risk of HIV infection for multigravida women may be increased due to blood transfusion during previous birth as a result of anemia. Likewise, this study revealed higher HIV prevalence compared to the recent nationwide HIV sentinel reports among ANC attendees in Ethiopia (2.2%) [10]. This could be because of sentinel reports are rough estimates which might not show the actual burden of the diseases, and this may underestimate HIV burden.

Moreover, the overall HIV prevalence among pregnant women in Ethiopia was higher than prevalence reports from Brazil (0.38%) [34] and Nigeria (3.0%) [35] equivalents. This might be due to Ethiopian pregnant women have little knowledge and attitude towards HIV and as well as the rate of HIV vertical transmission compared to Brazilians and Nigerians. Partly, it could be because of socio-economic or socio-cultural variations.

On the other hand, the overall HIV prevalence among pregnant women in Ethiopia was comparable with the report from Tanzania 5.6% [36]. Nevertheless, the pooled prevalence report was less than HIV prevalence reports from Zambia pregnant women (22.5%) [37]. This could be because of Zambia is one of the top eight countries with the highest HIV prevalence in Africa compared to Ethiopia [38]. Partly, it may be due to Ethiopian pregnant women have better information about HIV than the Zambian counterparts.

Besides, subgroup analysis was done based on different regions of Ethiopia to figure out the differences in the burden of HIV among pregnant women. As a result, a significant variation in HIV prevalence was observed across different regions. The pooled prevalence among subgroups indicated 9.50% (95% CI 7.76–11.23%) in Amhara, 4.80% (95% CI 3.12–6.49%) in Addis Ababa, 2.14% (95% CI − 0.54 to 4.82%) in SNNP and 4.48% (95% CI 2.56–6.41%) in Oromia region. However, recent sentinel surveys showed 2.8% in Amhara, 5.5% in Addis Ababa, 1.5% in SNNP and 1.3% in Oromia region [10]. The variation in HIV prevalence between this meta-analysis and sentinel survey could be due to the rough estimate nature of sentinel survey that might not be able to address the actual burden of the diseases explicitly in different regions of Ethiopia. Moreover, the difference in HIV burden among the regions in this meta-analysis might be due to variation in allocating budget, health care services, maternity care and HIV advocacy to different regions by the central government. The prevalence of HIV among pregnant women in Amhara region is twofold than other regions, indicating these people are at risk of developing HIV positive future generation unless an urgent intervention is implemented. Moreover, Amhara Public Health Institute (APHI) and regional researchers in the area has to declare an urge call for intervention with the regional governor as HIV is surging dramatically in the area.

In addition, pooled estimate of HIV-HBV co-infection was also assessed among pregnant women. Consequently, the overall pooled prevalence was 0.68% (95% CI 0.27–1.08%). In this study, HIV-HBV co-infection pooled estimate was less than prevalence reports from Rwanda pregnant women (4.1%) [39]. This could be due to less ongoing community level HBV transmission in Ethiopia compared to the Rwandan counterparts. Thus, HBV is adding layers of complexity to the most complex viral disease (HIV) among pregnant women in Ethiopia.

### Conclusions

The pooled prevalence of HIV infection in pregnant women was considerably high in Ethiopia. Specifically, high prevalence of HIV infection was determined in Amhara region compared to other regions. This study also assessed the burden of HIV-HBV co-infection. Therefore, national HIV prevention and interventional planning on pregnant women has to be based on HIV regional prevalence and its co-infection with HBV.

## Limitations

This study provides only prevalence information on HIV and HIV-HBV co-infection, so it fails to assess HIV and/or HBV vertical transmission as well as incidence rate. Furthermore, absence of data for some regions of Ethiopia may make difficulty to generalize the findings.

## Additional files

**Additional file 1.** General characteristics of included studies (n = 15).

**Additional file 2.** Sensitivity analysis of HIV prevalence among pregnant women in Ethiopia.

## Abbreviations

ANC: antenatal clinic
APHI: Amhara Public Health Institute
ART: antiretroviral therapy
CI: confidence interval
HBV: hepatitis B virus
HIV: human immunodeficiency virus
MTCT: mother to child transmission
SNNP: southern nation nationalities and peoples of Ethiopia
STI: sexually transmitted infections
WHO: World health organization
