# Supplementary material for: Seroprevalence of HIV among pregnant women in Ethiopia: a systematic review and meta-analysis
Source: BMC Res Notes. 2018 Dec 19;11:908. doi: 10.1186/s13104-018-4022-1 (PMC6299960; doi:10.1186/s13104-018-4022-1)
Supplement: Supplementary file 1 — Additional file 1. General characteristics of included studies (n = 15). [file 13104_2018_4022_MOESM1_ESM.docx]

**Additional file 1**

General characteristics of included studies (n = 15).

| Author/year | Study design | Study region | HIV diagnosis | Sample Size | HIV positive (%) | HIV-HBV positive (%) | Quality assessment |
| --- | --- | --- | --- | --- | --- | --- | --- |
| Asmamaw et al, 2013 [[17](#_ENREF_17)] | Cross sectional | Amhara | HIV-1/2 rapid test kits | 212 | 10.85 | __ | High quality |
| Desalegn et al, 2016 [[20](#_ENREF_20)] | Cross sectional | Addis Ababa | HIV-1/2 rapid test kits | 215 | 4.19 | 0.93 | High quality |
| Fissehatsion et al, 2017 [[22](#_ENREF_22)] | Cross sectional | Addis Ababa | HIV-1/2 rapid test kits | 403 | 5.20 | 0.50 | High quality |
| Mulu et al, 2007 [[25](#_ENREF_25)] | Cross sectional | Amhara | HIV-1 rapid test kits | 480 | 9.60 | __ | High quality |
| Endris et al, 2015 [[21](#_ENREF_21)] | Cross sectional | Amhara | HIV-1/2 rapid test kits | 385 | 11.20 |  | High quality |
| Metaferia et al, 2016 [[24](#_ENREF_24)] | Cross sectional | SNNP | HIV-1/2 rapid test kits | 269 | 5.20 | 0.74 | High quality |
| Desalegn et al, 2016 [[19](#_ENREF_19)] | Cross sectional | Oromia | HIV-1/2 rapid test kits | 202 | 3.50 | 0.50 | High quality |
| Melku et al, 2015 [[23](#_ENREF_23)] | Cross sectional | Amhara | HIV-1/2 rapid test kits | 300 | 10.33 | __ | High quality |
| Ramos et al, 2011 [[26](#_ENREF_26)] | Cross sectional | SNNP | *ELISA | 165 | 1.80 | 0.60 | High quality |
| Zenebe et al, 2014 [[28](#_ENREF_28)] | Cross sectional | Amhara | HIV-1/2 rapid test kits | 318 | 6.60 | 1.30 | High quality |
| Deme et al, 2016 [[7](#_ENREF_7)] | Cross sectional | SNNP | HIV-1/2 rapid test kits | 574 | 0.20 | __ | High quality |
| Mekonen et al, 2002 [[8](#_ENREF_8)] | Cross sectional | Oromia | ELISA | 224 | 12.10 | __ | High quality |
| Chegen et al, 2017 [[16](#_ENREF_16)] | Cross sectional | Oromia | HIV-1/2 rapid test kits | 1568 | 2.93 | __ | High quality |
| Cherinet et al, 2013 [[18](#_ENREF_18)] | Cross sectional | Oromia | HIV-1/2 rapid test kits | 7817 | 5.40 | __ | High quality |
| Schonfeld et al, 2017 [[27](#_ENREF_27)] | Prospective cohort | Oromia | HIV-1/2 rapid test kits | 296 | 2.00 | __ | High quality |

**Keys:** ELISA; Enzyme linked immunosorbent assay, HIV; Human immunodeficiency virus, SNNP; Southern nations nationalities and peoples of Ethiopia, * ELISA for detection of antibodies against both HIV-1 and HIV-2 followed by Western blot.
