# Supplementary material for: Seroprevalence of HIV among pregnant women in Ethiopia: a systematic review and meta-analysis
Source: BMC Res Notes. 2018 Dec 19;11:908. doi: 10.1186/s13104-018-4022-1 (PMC6299960; doi:10.1186/s13104-018-4022-1)
Supplement: Supplementary file 2 — Additional file 2. Sensitivity analysis of HIV prevalence among pregnant women in Ethiopia. [file 13104_2018_4022_MOESM2_ESM.docx]

**Additional file 2**

Sensitivity analysis of HIV prevalence among pregnant women in Ethiopia

| Excluded studies | Pooled prevalence(95% CI) | Excluded studies | Pooled prevalence(95% CI) |
| --- | --- | --- | --- |
| Asmamaw et al, 2013 | 5.45 (3.64, 7.26) | Ramos et al, 2011 | 6.05 (4.17, 7.93) |
| Desalegn et al, 2016 | 5.86 (4.00, 7.72) | Zenebe et al, 2014 | 5.68 (3.84, 7.53) |
| Fissehatsion et al, 2017 | 5.79 (3.93, 7.65) | Deme et al, 2016 | 5.99 (4.63, 7.36) |
| Mulu et al, 2007 | 5.46 (3.65, 7.26) | Mekonen et al, 2002 | 5.38 (3.58, 7.18) |
| Endris et al, 2015 | 5.37 (3.57, 7.16) | Chegen et al, 2017 | 6.03 (3.99, 8.06) |
| Metaferia et al, 2016 | 5.79 (3.93, 7.64) | Cherinet et al, 2013 | 5.78 (3.91, 7.65) |
| Desalegn et al, 2016 | 5.91 (4.04, 7.77) | Schonfeld et al, 2017 | 6.05 (4.16, 7.95) |
| Melku et al, 2015 | 5.44 (3.63, 7.25) |  |  |
| Combined | 5.74 (3.96, 7.53) | | |
